# Assessment of Sperm Binding Capacity in the Tubal Reservoir Using a Bovine Ex Vivo Oviduct Culture and Fluorescence Microscopy

**DOI:** 10.3390/mps4040067

**Published:** 2021-09-23

**Authors:** Miguel Camara Pirez, Simeng Li, Sabine Koelle

**Affiliations:** 1 Department of Health Sciences, School of Medicine, University College Dublin, D04 V1W8 Dublin, Ireland; miguel.camarapirez@ucdconnect.ie; 2 Systems Biology Ireland, School of Medicine, University College Dublin, D04 V1W8 Dublin, Ireland; simeng.li@ucdconnect.ie

**Keywords:** sperm, oviduct, sperm binding, sperm reservoir, fluorescence microscopy

## Abstract

Sperm binding within the oviductal sperm reservoir plays an important role for reproductive success by enabling sperm survival and maintaining fertilizing capacity. To date, numerous in vitro technologies have been established to measure sperm binding capacity to cultured oviductal cells or oviductal explants. However, these methods do not accurately represent the microenvironment and complex multi-molecular nature of the oviduct. In this paper, we describe a novel protocol for assessing sperm binding capacity in the tubal sperm reservoir using an ex vivo oviduct culture in the bovine model. This protocol includes the staining of frozen-thawed bovine spermatozoa with the DNA-binding dye Hoechst 33342, the co-incubation of stained sperm in closed segments of the oviduct and the visualization and quantification of bound spermatozoa by fluorescence microscopy. By generating overlays of multiple Z-stacks of randomly selected regions of interest (ROIs), spermatozoa bound in the sperm reservoir can be visualized and quantified within the 3D arrangement of the oviductal folds. This method, which is applicable to multiple species, can be used to assess individual sperm binding capacity in males for prognostic purposes as well as to assess the impact of diseases and medications on the formation of the sperm reservoir in the oviduct in humans and animals.

## 1. Introduction

The ability of spermatozoa to travel through the female reproductive tract, reach the oviduct and form the sperm reservoir is essential for successful fertilization in mammals. The formation of the sperm reservoir, which differs between species [1,2,3,4], is a complex process involving multiple receptors [5], glycans [6] and endocrine signaling [7] to prolong the survival of spermatozoa, select for acrosome-intact cells and limit the chances of polyspermy [8,9]. In most species, the majority of spermatozoa will bind to the isthmus of the oviduct [10,11,12], but some will also bind to the ampulla, the site of fertilization [13,14]. The oviductal glycans responsible for sperm binding in cattle include fucose [3], hyaluronan [15] and Lewis-a trisaccharide [16]. Following attachment to the oviductal cilia, spermatozoa undergo changes to stabilize their acrosomal and plasma membranes [17], adjusting the protein expression profile of the oviduct at the same time [18]. This bi-directional signaling between spermatozoa and the oviduct maintains sperm fertilizing capacity and delays capacitation [13,19], a series of physiological changes which prepare spermatozoa to fertilize the oocyte [20]. 

However, there is substantial variation in the ability of spermatozoa to bind to the oviduct as a result of variability in semen and sperm quality parameters among males and ejaculates [21,22]. It is therefore of great importance to assess and compare sperm binding capacity between males in order to maximize the likelihood of fertilization during breeding when using artificial insemination (AI). This is essential in bovine commercial farming, which aims to maximize profits while minimizing losses to time and labor [23]. To this end, studies have employed a number of in vitro technologies such as binding assays [24] as well as oviduct epithelium monolayers [25] and spheroid-like explants [26]. However, many of these methods fail to accurately reflect the in vivo microenvironment of the oviduct. Sperm binding assays often only measure binding capacity to a single glycoprotein or ligand and do not take the multi-molecular nature of sperm-oviduct interactions into account [27]. Monolayers of ciliated epithelial cells lack the 3D orientation of the oviductal folds [28], the response of secretory cells to sperm binding [29] and paracrine signaling between neighboring cells [30]. In a similar manner, spheroid explants do not accurately replicate the scaffolding anatomy of the oviduct [28]. 

In this protocol, we describe a method to quantitatively analyze sperm binding capacity in the bovine model using frozen-thawed spermatozoa and an ex vivo organ culture of the oviduct. The use of the intact oviduct maintains the complex microarchitecture of this organ and preserves the numerous multi-molecular sperm-oviduct interactions, including paracrine signaling, during the experimental analyses. Thus, this method is able to provide a more indicative measure of sperm binding capacity under near in vivo conditions. This protocol can be used in numerous species not only for the prognosis of sperm fertilizing capacity but also for the examination of the effects of medications and diseases on the formation of the sperm reservoir. 

## 2. Experimental Design

The experimental design is based on the staining of frozen-thawed bovine spermatozoa with the fluorescent DNA binding dye Hoechst 33342, and on the co-incubation of stained sperm in closed segments of the fallopian tube. The visualization and quantification of bound sperm is performed by fluorescence microscopy of the opened oviductal sample. By the creation of multiple Z-stacks and combination of overlays of randomly selected regions of interest, bound spermatozoa can be visualized and quantified.

### 2.1. Materials

- Semen straws (2–15 million spermatozoa/0.25 mL straw)
- Distilled water
- Sorensen’s buffer (see Reagents Setup)
- Hepes buffer (see Reagents Setup)
- Glutaraldehyde (25% solution), (Sigma-Aldrich, Arklow, Wicklow, Ireland; Cat. no.: G5882)
- KH_2_PO_4_ (Sigma-Aldrich, Arklow, Wicklow, Ireland; Cat. no.: P5655)
- Na_2_HPO_4_-2H_2_O (Sigma-Aldrich, Arklow, Wicklow, Ireland; Cat. no.: P71642)
- KCl (Sigma-Aldrich, Arklow, Wicklow, Ireland; Cat. no.: P3911)
- NaCl (Sigma-Aldrich, Arklow, Wicklow, Ireland; Cat. no.: S9888)
- MgCl_2_-6H_2_O (Sigma-Aldrich, Arklow, Wicklow, Ireland; Cat. no.: M9272)
- CaCl_2_-2H_2_O (Sigma-Aldrich, Arklow, Wicklow, Ireland; Cat. no.: 223506)
- D-(+)-Glucose (Sigma-Aldrich, Arklow, Wicklow, Ireland; Cat. no.: 49139)
- Hepes (Sigma-Aldrich, Arklow, Wicklow, Ireland; Cat. no.: H3375)
- Hoechst 33342 (ThermoFisher Scientific, Dublin, Ireland; Cat. no.: H3570)
- Fluo-4 AM (ThermoFisher Scientific, Dublin, Ireland; Cat. no.: F14217)
- Sterile disposable scalpels (ThermoFisher Scientific, Dublin, Ireland, Cat. no.: 11798343)
- Superfrost Plus slides (ThermoFisher Scientific, Dublin, Ireland; Cat. no.: 22-037-246)
- 1.5 mL Eppendorf tubes (Sigma-Aldrich, Arklow, Wicklow, Ireland, Cat. no.: T6649)
- Cover slips (Sigma-Aldrich, Arklow, Wicklow, Ireland, Cat. no.: BR470045)
- Pipette tips (P20), (Starlab, Milton Keynes, UK; Cat. no.: S1110-3710)
- Pipette tips (P200), (Starlab, Milton Keynes, UK; Cat. no.: S1111-1716)
- Pipette tips (P1000), (Starlab, Milton Keynes, UK; Cat. no.: S1111-6701)
- Lint-free tissue wipes (VWR, Dublin, Ireland, Cat. no.: 115-0202)

### 2.2. Equipment

- Olympus BX51 Fluorescence Microscope (Olympus, Hamburg, Germany)
- DP71 camera (Olympus, Hamburg, Germany)
- Olympus CKX3-SLP phase contrast filter (Olympus, Hamburg, Germany)
- Olympus Filter Cube (Edmund Optics, York, UK; Cat. no.: 86-371)
- DAPI dichroic filter (Edmund Optics, York, UK; Cat. no.: 86-330)
- MyBlock™ Mini Dry Bath (Benchmark Scientific, Sayreville, NJ, USA; Cat. no.: BSH200)
- Heating block (Benchmark Scientific, Sayreville, NJ, USA; Cat. no.: BSH100-15)
- Thermometer (ThermoFisher Scientific, Dublin, Ireland, Cat. no.: 15350684)
- Dissecting scissors (ThermoFisher Scientific, Dublin, Ireland, Cat. no.: 12847622)
- Neubauer counting chamber (ThermoFisher Scientific, Dublin, Ireland; Cat. no.: 02-671-54).
- Centrifuge 5702 (Eppendorf, Stevenage, UK; Cat. no.: 5702000329)
- Dewar (KGW Isotherm, Karlsruhe, Germany; Cat. no.: 1211)
- General application forceps (30 cm) (ThermoFisher Scientific, Dublin, Ireland, Cat. no.: 16-100-107)
- Micropipette (P2.5), (Eppendorf, Stevenage, UK; Cat. no.: 3123000012)
- Micropipette (P20), (Eppendorf, Stevenage, UK; Cat. no.: 3124000032)
- Micropipette (P100), (Eppendorf, Stevenage, UK; Cat. no.: 3124000075)
- Micropipette (P1000), (Eppendorf, Stevenage, UK; Cat. no.: 3123000063)

## 3. Procedure

### 3.1. Collection and Preparation of Fallopian Tubes (1–2 hrs)

**Figure 1:**
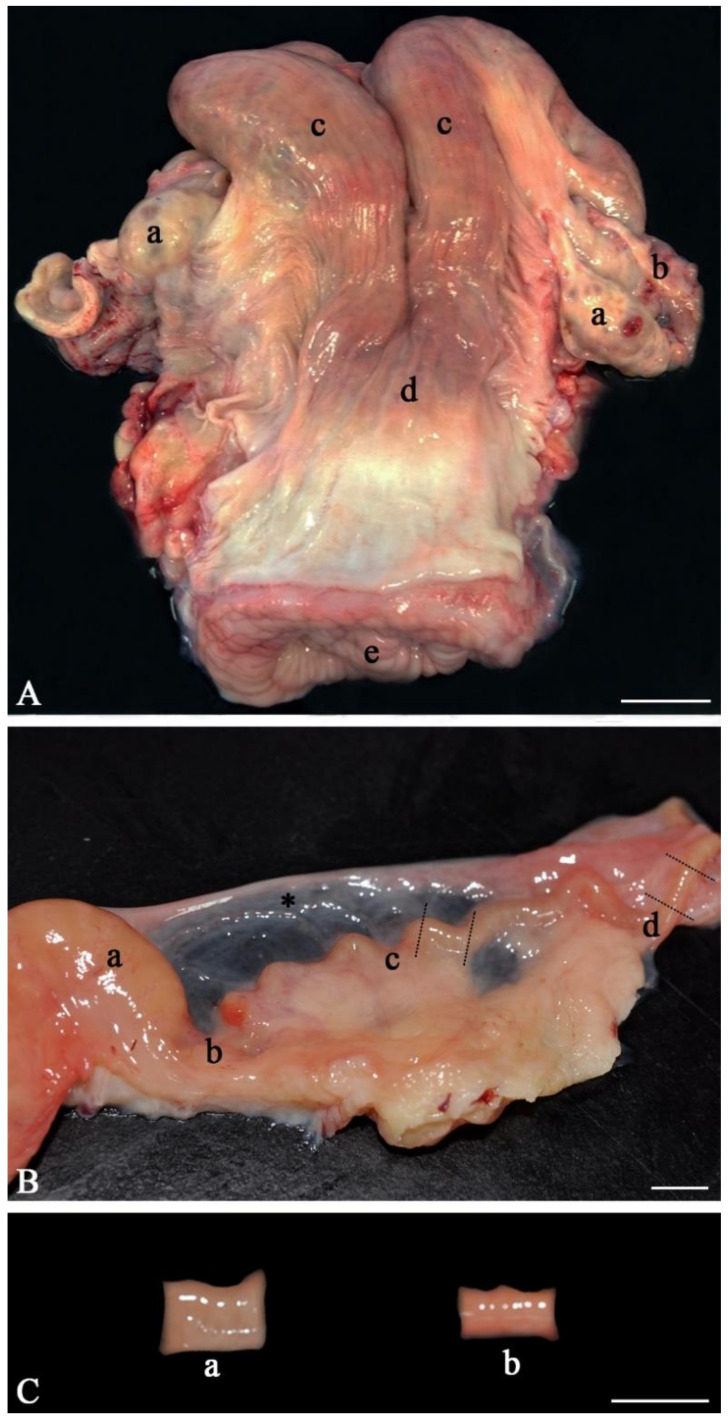
Anatomy of the bovine female genital tract. (**A**) Each tract consists of two ovaries (a), oviducts (b), uterine horns (c), a uterine body (d), and a cervix (e). (**B**) Each oviduct is connected to an ovary (a) through an infundibulum (b). The infundibulum catches the cumulus–oocyte complex (COC) after ovulation and leads the COC into the ampulla, where fertilization occurs (c). The isthmus (d) transports the early embryo into the uterus. The oviduct is surrounded by the mesosalpinx (*). The dotted lines indicate the cutting sites during dissection. (**C**) Segments dissected from the ampulla (a) and isthmus (b). (Scale bars: A = 5 cm, B and C = 1 cm).

**Figure 2:**
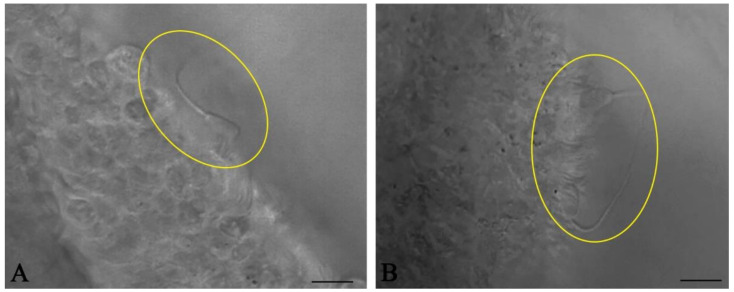
Live cell imaging of sperm binding in the bovine oviduct. Spermatozoa (circles) bind to the ciliated epithelial cells of both the ampulla (**A**) and isthmus (**B**). (Scale bars: 10 µm).

1. Collect heifer/cow female reproductive tracts from an abattoir immediately after slaughter. Wrap them in a plastic bag and keep them on ice (4 °C) during transport back to a lab;
2. Anatomically inspect the whole female genital tract. Only use healthy genital tracts (Figure 1A). Exclude genital tracts with signs of inflammation such as reddening of the mucosa and accumulation of fluid or pus. Be aware that an infection/inflammation in the uterus also means inflammation of the oviduct as all parts of the genital organs are in contact with the fluid of the genital tract, which is moved up and down by smooth muscle contractions. Clearly distinguish between the different parts of the oviduct (infundibulum, ampulla, isthmus, Figure 1B). Diagnose the stage of the estrous cycle by macroscopic assessment of the ovaries, uterine body and cervix. For example, features of the diestrus stage include the presence of a corpus luteum on the ovary as well as a flaccid muscle tone, a lack of secretions and a closed cervix. In contrast, estrus is characterized by the presence of a Graafian follicle on one of the ovaries, as well as a high muscle tone, high amount of secretions and an open cervix;
3. With anatomical scissors, carefully separate both oviducts from the rest of the tract and pin them onto a dissection board with the pins on both ends of the tube;
4. Using a scalpel, dissect and remove the surrounding mesosalpinx from the oviduct;
5. Carefully cut the isthmus or the ampulla in 1 cm pieces (Figure 1C). As shown by live cell imaging in the native oviduct, sperm binding occurs both in the ampulla (Figure 2A) and in the isthmus (Figure 2B). In the isthmus, the lumen is narrower than in the ampulla so that it is advisable to use surgical eye scissors to open it up. Always use the same part of the ampulla or isthmus (e.g. the middle third of the isthmus or the junction between ampulla and isthmus) for comparative analyses of sperm binding in individual males or before and after specific treatments. Our studies revealed that—if more tissue is needed—the same sites of right and left oviducts can be used.
6. Store the samples in Hepes buffer (4 °C) until stage 3.6.

### 3.2. Sperm Thawing (5 min)

1. Transfer the semen straws (250 µL) from their storage tank to a liquid nitrogen-filled dewar using a suitable forceps;
2. Prepare a water thawing bath with a temperature of 39 °C and transfer the semen straw to the water bath for 10 s;
3. Remove the straw from the water bath and wipe it with lint-free tissue to remove excess water;
4. Using scissors, cut the semen straw at the sealed end and place the newly exposed opening in a pre-heated (37 °C) 1.5 mL Eppendorf tube. Cut the semen straw below the cotton plug so that the semen is transferred to the Eppendorf tube.

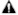
**CRITICAL STEP:** Ensure that the spermatozoa are kept at a constant temperature of 37 °C until the fixation step. Major deviations in temperature will reduce sperm motility and binding capacity.

### 3.3. Motility Analysis and Semen Washing (5 min)

1. Transfer 7 µL of semen to a non-adhesive glass slide, cover slip the semen and examine the spermatozoa using a phase contrast microscope with a 20× or 40× lens;
2. Estimate the total post-thaw motility of the spermatozoa. Only straws with >60% motility should be included in subsequent analyses;
3. Wash the semen by suspending it in 1 mL Hepes buffer and centrifuge for 2 min at 200 rcf;
4. Discard the supernatant and resuspend the resulting sperm pellet in 30 µL Hepes buffer;
5. Estimate the post-wash motility of the spermatozoa. The overall motility should be >60%.

### 3.4. Adjustment of Concentrations (5 min)

Due to the variability in sperm concentration in semen straws, it is necessary to equilibrate samples prior to co-incubation with the oviduct. This is of huge importance for the comparison of different groups of sperm which have undergone different treatments i.e., conventional and sex-sorted spermatozoa.

1. In an Eppendorf tube, dilute 2 µL of post-wash semen sample in 8 µL distilled water to create a 5× dilution;
2. Load all 10 µL of the diluted sample into a cover slipped Neubauer counting chamber;
3. Focusing on the center section of the grid, count the spermatozoa in five squares (top left, top right, bottom left, bottom right, and center square);
4. Calculate the concentration of the post-wash samples using the following formula:

$$Concentration\;\left(sperm/µL\right)=number\;of\;spermatozoa\;in\;5\;squares\times 5\times 5\times 10,000$$

5. Repeat steps 2–5 with all samples;
6. Adjust the concentration of the samples by diluting accordingly with Hepes buffer. Normally, the final concentration should be 7000–8000 sperm/µL.

### 3.5. Sperm DNA Staining (15 min)

1. Add Hoechst 33342 to the sperm samples to a final concentration of 20 µM and incubate for 10 min at 37 °C;

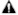
**CRITICAL STEP:** From this point on, keep the sperm samples in the dark to avoid photobleaching of Hoechst 33342.

2. Transfer 3–5 µL of the samples to a non-coated slide and cover slip. Image five regions of interest (ROIs, top left, bottom left, top right, bottom right and center) using a 20× lens under both phase contrast and fluorescent light using a DAPI filter. At least 200–400 spermatozoa should be imaged (see Section 4.2 for additional details);
Calculate the staining efficiency using the following formula:

$$Staining\;efficiency\;\left(\%\right)=\frac{number\;of\;spermatozoa\;visualised\;under\;fluorscence}{number\;of\;spermatozoa\;visualised\;under\;phase\;contrast}\;$$

4. Repeat steps 1–3 for all samples. Only include samples with a staining efficiency of >85% for further analyses.

### 3.6. Co-Incubation of Spermatozoa with Oviduct (12 min)

1. Remove a 1 cm segment of oviduct from the Hepes buffer and place it in on a heated plate or in an incubator (37°);
2. Using the smallest pipette tip possible to minimize damage to the oviductal epithelium, pipette 30 µL of a sperm sample and keep it at hand;
3. Using a forceps, gently open one end of an ampulla segment and deposit all 30 µL of the sperm sample into the middle region of the oviductal piece;
4. Incubate the ampulla segments for 10 min at 37 °C to allow the spermatozoa to bind to the oviductal epithelium.

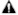
**CRITICAL STEP:** To accurately compare sperm binding capacity between different males, pieces from the same oviduct must be used to eliminate the variability of the individual female genital tract.

5. Gently rinse the oviduct in PBS to wash off unbound sperm;
6. Gently transfer the tubal segment to a Falcon tube filled with 2.5% glutaraldehyde in Sorensen’s buffer solution. The bound spermatozoa will be fixed to the oviductal epithelium within 10 min;

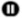
**PAUSE STEP:** The oviductal samples with sperm can be stored for up to 24 hrs at 4 °C before analysis.

7. Repeat steps 1–6 for all samples.

### 3.7. Imaging of Spermatozoa (15 min per Sample)

1. Open the fixed oviduct segment longitudinally;
2. Gently place the segment on a glass slide with the inner lining of the ampulla facing upwards;
3. Place the segment under a fluorescence microscope and visualize the bound spermatozoa under a DAPI filter using a 20× lens. Image the spermatozoa in at least 5 ROIs or, alternatively, create consecutive images of the whole segment;
4. Due to the 3D arrangement of the oviductal folds, it is necessary to finely adjust the objective to focus on various Z-planes at each region of interest;
5. Repeat steps 1–4 for all samples.

### 3.8. Image Overlaying and True Sperm Number (20–60 min)

1. Open Adobe Photoshop (Adobe Inc, San Jose, CA, USA);
2. Import all the Z-plane images for the same region of interest as stacks (File → Scripts → Load files into stacks). This will convert the images into layers;
3. Select all layers and overlay them (Edit → Auto-blend layers);
4. In the new window, select Stack Images as the blend method. Ensure “Seamless tones and colors” and “Content aware fill transparent areas” are selected;
5. Click OK. The software will detect which areas in each Z-stack are in focus and combine the focused areas into a master image. This process will take longer the more stacks are present. 12–14 Z-stacks per region of interest should be sufficient (see Section 4.3 for additional details);
6. Count the number of fluorescent bound spermatozoa in master image and calculate the average number of spermatozoa per tubal sample;
7. Since not all spermatozoa will fluoresce, the true number of bound spermatozoa can be calculated as follows:

$$Number\;of\;bound\;spermatozoa=\frac{Number\;of\;fluorescent\;bound\;spermatozoa}{Staining\;efficiency}$$

8. Once the number of bound spermatozoa per region/segment has been calculated, the sperm binding capacity between different males can be compared.

**Note** **1:** Readily available alternatives to Photoshop such as ImageJ may also be used for the overlaying of images.

**Note** **2:** As shown by preliminary studies using transmission electron microscopy, scanning electron microscopy and histochemistry, the oviductal pieces perform all the signals and secretions similar to in vivo up to a duration of 3 h. Consequently, the experimental procedure should be finished within a time frame of 3 h.

## 4. Expected Results

### 4.1. Sperm-Oviduct Interactions and Capacitation in Sperm Stained with Hoechst 33342

**Figure 3:**
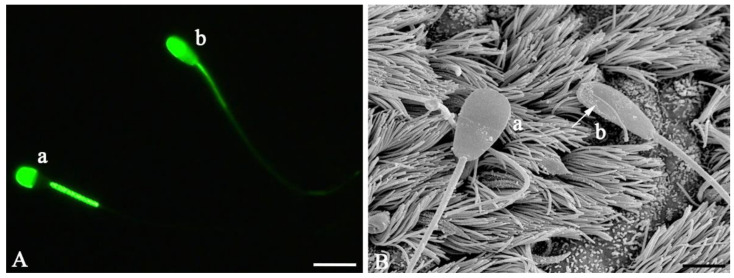
Evidence of capacitation in bovine spermatozoa before and after sperm binding in the ampulla. (**A**) Fluorescence microscopy of bovine spermatozoa using Fluo-4 AM. Capacitated sperm (a) present with a fluorescence-free band in the post-acrosomal region of the sperm head, which is not observed in uncapacitated sperm (b). (**B**) Scanning electron micrograph of capacitated sperm with a smooth plasma membrane (a) and uncapacitated sperm (b) revealing numerous protein vesicles on the surface of the head. This sperm also shows a defect in the plasma membrane (arrow). (Scale bars: A = 10 µm, B = 5 µm).

In a first step it was confirmed that capacitation can place in the ampulla and after Hoechst staining. Capacitation is a process which takes place during the migration through the female genital tract and involves morphological and biochemical changes such as the removal of proteins from the sperm surface [31]. As this reaction is important for the fertilizing ability of sperm [32] we applied scanning electron microscopy and fluorescent staining with Fluo-4 to visualize capacitation both in stained and unstained sperm and both before and after binding capacitation occurred. Figure 3A shows a capacitated sperm (a) with a prominent fluorescent acrosome and a fluorescent-free band in the post-acrosomal region and a non-capacitated sperm which shows fluorescence in the whole head. Figure 3B shows a capacitated sperm (a) which is characterized by a smooth acrosomal region compared to a non-capacitated sperm (b) which has numerous protein vesicles attached to the plasma membrane of the head.

### 4.2. Staining Efficiency

**Figure 4:**
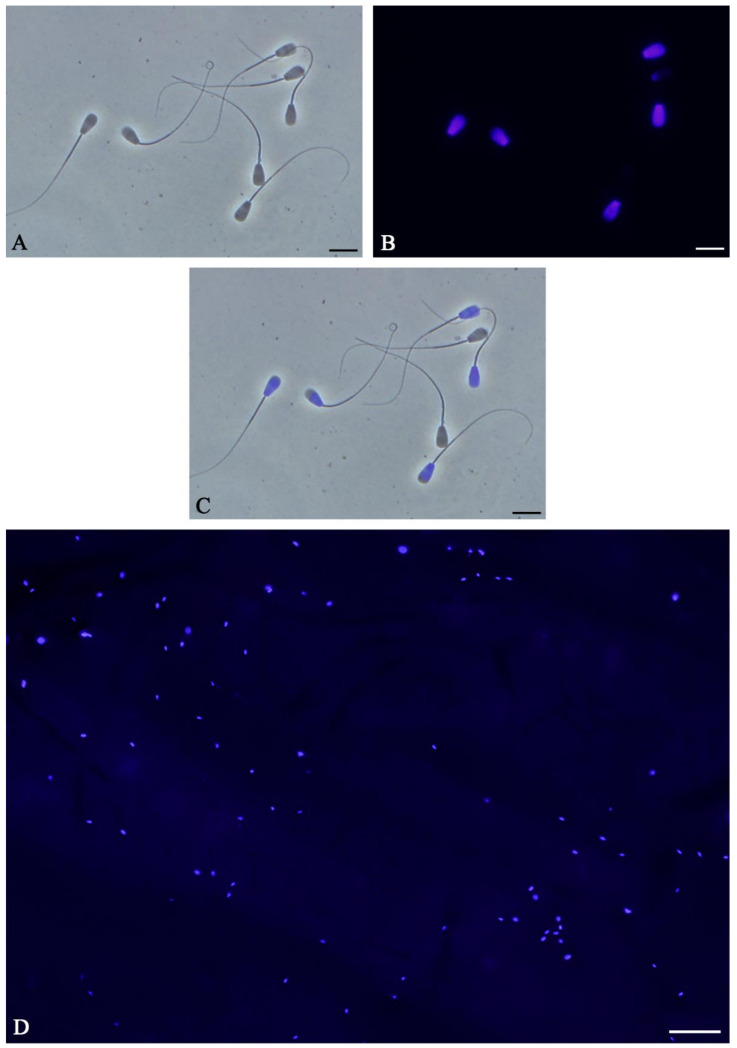
Hoechst 33342 staining of bovine spermatozoa to measure binding capacity in the oviduct. By combining phase contrast (**A**) and fluorescent images (**B**) into overlays (**C**), it is possible to identify spermatozoa which have been successfully stained. In this image, five of seven spermatozoa have taken up the dye. (**D**) Master image of bound spermatozoa in the oviduct. Each fluorescent light blue spot corresponds to a single spermatozoon. (Scale bars: A–C: 10 µm; D = 100 µm).

Following incubation with Hoechst 33342, it is crucial to calculate the staining efficiency of the procedure as Hoechst 33342 will not readily enter all spermatozoa. It is recommended that at least 85% of spermatozoa fluoresce under blue light in a similar fashion as shown in Figure 4. By combining phase contrast (Figure 4A) and fluorescent (Figure 4B) images into an overlay (Figure 4C), it is possible to accurately determine the frequency of spermatozoa which have been successfully stained. If the staining efficiency in at least 200 spermatozoa is less than 85%, it is recommended to use a higher concentration of Hoechst 33342. Concentrations as high as 112 µM have been reported [33]. Alternatively, incubation times can be increased in 5 min increments up to 20 min.

### 4.3. Sperm Binding Capacity

If enough Z-stack images were taken (at least 12–14), Photoshop is able to generate a master image or overlay of each ROI in which most, if not all, spermatozoa are in focus and can be visualized (Figure 4D). By creating an overlay, the number of fluorescent, bound spermatozoa within that region can be counted, with each fluorescent light blue spot corresponding to a single spermatozoon. Make sure that the spots you include in the quantitative analysis have the typical size of the sperm head, which is, for example, 8 µm in the bovine and 6 µm in humans.

After implementing the staining efficiency, the average number of spermatozoa per tubal segment can be compared between different males. As the sperm concentrations have been adjusted and the same oviduct is being used for multiple males, the only factor responsible for differences in the number of spermatozoa per region/segment is the difference in sperm binding capacity. According to our experience, the functional integrity of the oviduct in the ex vivo culture as described above is maintained up to 2.5 h after removal. Thus, this method is a good indicator for sperm binding capacity under near in vivo conditions.

## 5. Discussion

The sperm binding assay in the ex vivo organ culture of the oviduct is a powerful tool to analyze sperm binding capacity of individual males under near in vivo conditions. This binding assay using an ex vivo organ model has several advantages over traditional cultures because it is able to account for the complex and multi-molecular nature of sperm-oviduct interactions. In contrast, most in vitro binding assays only measure affinity to a single molecule such as fucose [34]. Further to that, this model maintains the 3D orientation of the oviductal folds as well as the paracrine signaling between neighboring epithelial cells while monolayers and spheroids do not [25,26]. As both the ampulla and the isthmus are involved in formation of the sperm reservoir, both parts can be used for comparative sperm binding analysis [35,36,37]. Our preliminary studies confirmed that there are no differences between ampulla and isthmus regarding sperm behavior and sperm movement patterns after sperm binding. Suarez et al., (2006) reported that after ovulation most sperm in the ampulla were located near the cumulus–oocyte complex [13]. Consequently, the ampulla is an important site for investigation as spermatozoa bound in the ampulla are those who will be the first spermatozoa to reach the oocyte after ovulation. However, this protocol also has limitations. Spermatozoa must be prepared to remain in the sperm reservoir for 3–4 days in most mammals, including humans and bovine [38]. Unfortunately, the organ culture in this model remains viable only for a few hours. Therefore, it cannot be used to measure binding capacity over longer periods of time. Additionally, the protocol includes additional dilution steps so that individual spermatozoa within the 3D arrangement of the oviductal folds can be identified. Dilutions are known to remove decapacitation factors from the sperm surface [39]. If these factors are removed, capacitation processes such as the acrosome reaction may be prematurely triggered, causing a loss of sperm surface proteins and reducing binding capacity [40]. However, as many of these proteins are already removed uniformly between males during the process of cryopreservation of spermatozoa, this assay can be considered as representative of sperm binding in the oviduct when using frozen-thawed spermatozoa for insemination.

## 6. Conclusions

In summary, the established sperm binding assay is a unique and robust method for comparing tubal sperm binding capacity of individual males. It only requires basic laboratory facilities and low-cost materials. As sperm binding in the oviduct is an essential prerequisite to maintaining fertilizing ability and ensuring sperm survival, it is a valuable prognostic marker for fertility. It also enables the investigation of the effects of diseases and medications on sperm binding capacity thus contributing to important insights on sperm behavior and function within the oviduct.

## 7. Reagents Setup

### 7.1. Preparation of Hepes Stock Solutions

1. Add 10.16 g MgCl_2_-6H_2_O to 50 mL distilled water (final concentration = 1 M);
2. Add 7.35 g CaCl_2_-2H_2_O to 50 mL distilled water (final concentration = 1 M).

### 7.2. Preparation of Hepes Buffer

1. Add the reagents in Table 1 to 1 L distilled water;
2. Adjust the pH of the solution to 7.4 using NaOH;
3. Store the Hepes buffer at 4 °C for up to a month.

### 7.3. Preparation of Sorensen’s Buffer

1. Prepare Solution A by adding 0.91 g KH_2_PO_4_ per 100 mL distilled water;
2. Prepare Solution B by adding 1.19 g Na_2_HPO_4_-2H_2_O per 100 mL distilled water;
3. Mix Solutions A and B in a 1:4 (*v*/*v*) ratio. This mixture is Sorensen’s buffer;
4. Store the Sorensen’s buffer at 4 °C for up to a month.

### 7.4. Preparation of Fixing Solution

1. Add 1 mL of 25% glutaraldehyde solution per 6.25 mL Sorensen’s buffer;
2. Store fixing solution at 4 °C for up to a month.

## Figures and Tables

**Table 1:** Reagents for Hepes buffer solution.

Chemical	Quantity	Final Concentration
KCl	0.418 g	5.6 mM
NaCl	7.970 g	136.4 mM
MgCl_2_-6H_2_O (1 M)	1.000 mL	1 mM
CaCl_2_-2H_2_O (1 M)	2.200 mL	2.2 mM
D-(+)-Glucose	1.980 g	11 mM
HEPES	2.380 g	10 mM
